# Ribosomal Protein L15 is involved in Colon Carcinogenesis

**DOI:** 10.7150/ijms.34386

**Published:** 2019-08-06

**Authors:** Zhixiong Dong, Hongyu Jiang, Shuangshuang Liang, Yajie Wang, Wei Jiang, Changjun Zhu

**Affiliations:** 1 Key Laboratory of Laboratory Medicine, School of Laboratory Medicine and Life Science, Wenzhou Medical University, Wenzhou, Zhejiang 325035, China;; 2 Tianjin Key Laboratory of Animal and Plant Resistance, College of Life Sciences, Tianjin Normal University, Tianjin 300387, China;; 3 Key Laboratory of Molecular and Cellular Systems Biology, College of Life Sciences, Tianjin Normal University, Tianjin 300387, China;; 4 State Key Laboratory of Molecular Oncology, Cancer Institute and Hospital, Chinese Academy of Medical Sciences and Peking Union Medical College, Beijing 100021, China;; 5 AstraZeneca Pharmaceutical Co Ltd, Xi'an, 710100, China.

**Keywords:** RPL RPL15, nucleolus, ribosome biogenesis, colon cancer, apoptosis 15, nucleolus, ribosome biogenesis, colon cancer, apoptosis

## Abstract

Ribosomal biogenesis is responsible for protein synthesis in all eukaryotic cells. Perturbation of ribosomal biogenesis processes can cause dysfunctions of protein synthesis and varieties of human diseases. In this study, we examine the role of RPL15, a large ribosomal subunit protein, in human colon carcinogenesis. Our results reveal that RPL15 is remarkably upregulated in human primary colon cancer tissues and cultured cell lines when compared with paired non-cancerous tissues and non-transformed epithelium cells. Elevated expression of RPL15 in colon cancer tissues is closely correlated with clinicopathological characteristics in patients. We determine the effects of RPL15 on nucleolar maintenance, ribosomal biogenesis and cell proliferation in human cells. We show that RPL15 is required for maintenance of nucleolar structure and formation of pre-60S subunits in the nucleoli. Depletion of RPL15 causes ribosomal stress, resulting in a G1-G1/S cell cycle arrest in non-transformed human epithelium cells, but apoptosis in colon cancer cells. Together, these results indicate that RPL15 is involved in human colon carcinogenesis and might be a potential clinical biomarker and/or target for colon cancer therapy.

## Introduction

Ribosome is an intracellular organelle that is responsible for translating genetic information encoded in messenger RNAs (mRNAs) into functional proteins to sustain cell growth, proliferation, differentiation and metabolism, etc. Eukaryotes have 80S ribosome consisting of a large (60S) and a small (40S) subunit. The 60S subunit consists of a 5S rRNA, a 5.8S rRNA, a 28S rRNA and about 49 proteins (RPLs). The 40S subunit is composed of an 18S rRNA and approximately 33 proteins (RPSs)^1^. In addition to protein synthesis, ribosomal proteins have been demonstrated to take part in several other extraribosomal functions. For example, several ribosomal proteins have been found to play a role in DNA repair, transcription, RNA processing and apoptosis^2^, ^3^.

Given the important function of ribosome and ribosomal proteins (RPs) in cells, ribosomal dysfunction would result in a variety of diseases 4-7. It has been shown that plenty of birth defects and anemia were closely associated with mutations or deletions of ribosomal proteins. There are abundant genetic and experimental evidences demonstrate that Diamond-Blackfan anemia (DBA), a dominant autosomal bone marrow failure syndrome, is due to mutations in ribosomal genes including RPS19, RPS24, RPL5, RPL11, and RPS29, etc^8^-^12^. In addition, it was also reported that ribosomal protein disorders associated with malignancies. DBA patients have higher risk of cancer than the general population, specifically a higher risk of developing acute myelocytic leukemia (AML), osteosarcoma, or colon cancer^13^. Changes in the expression levels of RPs in cancer are common^14^. For example, RPS2 is found to be overexpressed in liver cancer^15^, and RPL7A, RPL19, RPL37 are overexpressed in prostate cancer^16^, ^17^. In addition, response to ribosome stress, RPL5, RPL11 and 5S rRNA have been demonstrated to interact with Mdm2 and inhibit Mdm2 E3 ligase activity, thereby increase p53 protein stability and transcriptional activity, thus activating p53-dependent cell cycle checkpoints^18^-^20^. RPL11 and RPL5 have been referred as tumor suppressors, and their deletion or mutation were found in several cancers^21^-^23^.

Previous studies reported that Ribosomal protein L15 (RPL15), a component of 60S subunit, not only participates in ribosomal assembly but also regulates pre-rRNA processing^12^, ^24^. RPL15 was found to be dysregulated in many types of diseases. For instance, RPL15 was detected existing depletion in a DBA patient^12^. However, the correlation profiles of RPL15 with cancer are different depending on the type of cancer. Wang et al. found that RPL15 is overexpressed in esophageal cancer^25^. Additionally, it was reported that upregulation of RPL15 is associated with cell proliferation in gastric cancer^23^. However, other studies revealed that RPL15 is downregulated in cutaneous squamous cell carcinoma and pancreatic ductal adenocarcinoma^26^, ^27^, which were contrast to the former two studies. To date, little is known about the role of RPL15 in colon cancer.

In this study, we investigate the role of RPL15 in colon carcinogenesis. We find that RPL15 is overexpressed in colon cancer cells and tissues, and the expression of RPL15 is closely associated with colon cancer carcinogenesis. Furthermore, we demonstrate that depletion of RPL15 induces apoptosis in colon cancer cells, but cell cycle arrest in non-transformed RPE1 cells. These results support the potential value of RPL15 as a therapeutic target in colon cancer treatment.

## Materials and Methods

***Cell culture, transfection and drug treatment*.** Human cervical carcinomas HeLa cells, human colon carcinoma HCT116 cells and RPE1 (hTERT-RPE1) cells were purchased from ATCC. HeLa and HCT116 cells were cultured in DMEM (HyClone) supplemented with 10% fetal bovine serum (FBS) (HyClone). RPE1 cells were cultured in DMEM: F-12 (1:1) (HyClone) containing 10% FBS. All cells were cultured at 37°C in 5% CO_2_. Plasmid/siRNA transfection was conducted with Lipofectamine 3000 and/or RNAiMAX Reagent (Life Technologies Inc) according to manufacturer's protocol. In brief, HeLa, HCT116 and RPE1 cells were plated in 96-well plates (3000 cells/well), 24-well plates (2×10^4^ cells/well) or 6-well plates (1×10^5^ cells/well) for 16 h at 37°C before transfection. Then, cells were incubated with indicated plasmids/siRNAs plus transfection reagent mixture in medium for 24 h and then changed into fresh medium.

***Plasmids, siRNAs and antibodies.*** The full-length coding region of RPL15 cDNA was generated by PCR and subcloned into EcoR I-Sal I sites of the mammalian expression vector pEGFP-C2. The construct was fully sequenced. siRNAs specific targeting to RPL15 (1#: 5'-UGGUGUUAACCAGCUAAAGdTdT-3'; 2#: 5'-UCCAGGAGCUAUGGAGAAAdTdT-3') were synthesized by Genepharma (Shanghai, China). Polyclonal rabbit α-RPL15 were generated against peptide of CSRRAAWRRRNTLQLHRYR. Mouse α-UBF (#sc-13125), mouse α-p53 (#sc-126), rabbit α-p21 (#sc-397) and mouse α-nucleolin (#sc-8031) were purchased from Santa Cruz Biotechnology. Mouse α-fibrillarin (#ab4566) was purchased from Abcam. Mouse α-BrdU (#5292) and mouse α-RPS6 (#2317) were purchased from Cell Signaling Technology. Mouse α-RPL11 (#37-3000) was purchased from Life Technologies Inc. Rabbit α-H2AX (#3522-1) was purchased from Epitomics. Mouse α-Bip (#27033) was purchased from signalway antibody. Mouse anti-α-tubulin antibody (T5168) was purchased from Sigma-Aldrich. All secondary antibodies were obtained from Life Technologies Inc.

Tissue samples. Colon cancer specimens and adjacent histologic normal tissues were obtained from 25 patients who underwent surgery in Department of Cancer Research Institute, Cancer Affiliated Hospital of Xinjiang Medical University, Urumqi, China. All specimens were immediately frozen in liquid nitrogen and stored at -80°C until use. The patients' medical data and lifestyle for cancer risk factors (e.g. family history of cancer) were documented with informed consent.

***Preribosome preparation*.** Nuclear extracts were fractionated and preribosome preparation was performed as described previouslywith minor modifications^28^. In brief, HeLa cells were swollen in ice-cold hypotonic lysis buffer (10 mM Tris [pH 7.4], 10 mM KCl, 2 mM MgCl_2_, 0.05% Triton X-100, 1 mM EGTA, 1 mM DTT, 40 mg/ml of phenylmethylsulfonyl fluoride, and 10 mg/ml of protease inhibitor cocktail). The nuclei pellet was collected by centrifugation at 500×g for 5 min. The nuclear lysate was extracted in extraction buffer (25 mM Tris (pH 7.5), 100 mM KCl, 1 mM DTT, 2 mM EDTA, 0.1% NP-40, 1 mM NaF, 40 mg/ml of phenylmethylsulfonyl fluoride, 10 mg/ml of protease inhibitor cocktail and 0.1U/ml of RNasin (Promega) and sonicated. The nuclear lysate was overlaid on 10 to 30% (wt/wt) sucrose gradients in preribosome buffer (25 mM Tris (pH 7.5), 100 mM KCl, 1 mM DTT and 2 mM EDTA) and centrifuged at 36,000 rpm for 3 h at 4°C in a Beckman SW41Ti rotor. The gradients were collected downward and the absorbance of each fraction was measured at 260 nm using a spectrophotometer.

***Cell proliferation (MTT) assay, cell cycle analysis and BrdU incorporation assay.*** For cell proliferation (MTT) assay, HCT116 or RPE1 cells were plated in 96-well plates (3000 cells/well) for 16 h. Cells were then transfected with or without RPL15 siRNA using RNAiMAX Transfection Reagent for 24 h. After transfection, cells were changed into fresh medium and determined by MTT (3-(4,5-dimethylthiazol-2-yl)-2,5-diphenyltetrazolium bromide) analysis at indicated times as described previously^29^.

For cell cycle analysis, HCT116 or RPE1 were fixed in 70% ethanol/30% phosphate-buffered saline (PBS) for 1 h at -20°C. After fixation, cells were washed once with PBS, resuspended, and incubated in propidium iodide (PI) buffer (60 μg/ml PI and 0.1 mg/ml RNase A) for 45 min at room temperature. Flow cytometry was conducted on at least 5,000 cells per condition using a FACSort and CellQuest version 3.3 (BD Biosciences). Cell cycle profiles were processed and analyzed using FlowJo version 6.4.7 (Tree Star, Ashland, OR).

BrdU incorporation was performed as described previously^28^. Cells were fixed and immunostained with mouse α-BrdU. The percentages of BrdU positive cells were scored (>1000 cells) using a fluorescence microscope.

***Immunoblotting and immunofluorescence analyses*.** Cells or cells transfected with indicated plasmid and/or siRNA or treated with indicated drug were harvested or fixed for immunoblotting or immunofluorescence analysis as previously described^30^. In brief, for immunoblotting, cells harvested and lysed in 1% Nonidet P-40 buffer^31^. Cell lysates with equal amounts of total protein were subjected to SDS-PAGE, transferred to PVDF membrane and then immunoblotted with corresponding antibodies. For immunofluorescence analysis, cells grown on glass coverslips were fixed and immunostained with indicated antibodies. Cells were photographed using a NIKON fluorescence microscope. Immunofluorescent area was measured using Image-Pro Plus 7.0 (Media Cybernetics). Pearson's correlation coefficients (R value) were calculated using Image-Pro Plus 7.0^32^.

***RNA isolation and Real-time RT-PCR.***Total RNA was isolated using the Trizol reagent (Invitrogen) following manufacturer's instructions. One microgram RNA was used for cDNA synthesis using a reverse transcriptase reaction kit (Promega) and quantitative real-time PCR was performed on an ABI Prism 7000 Sequence Detection System (Applied Biosystems), using SYBR Green (TIANGEN BIOTECH) as a dsDNA-specific fluorescent dye. β-actin was used for standardizing 47S rRNA level. Amplification primers were 5'-GATTCGTGTTCGCCGTGGT-3' and 5'- TGCTTGTGGACTGGTTTGG-3' for RPL15, and 5'-TCGTGCGTGACATTAAGGAG-3' and 5'-ATGCCAGGGTACATGGTGGT-3' for β-actin. Data were analyzed by using the 2^-ΔΔCt^ method^33^. All results represent means± standard deviations of three independent experiments.

***Separation of the cytoplasm and nucleus.***HeLa cells were swollen in ice-cold hypotonic buffer (10 mM Hepes-NaOH [pH 7.5], 10 mM NaCl, 2 mM MgCl_2_, 1 mM EDTA) and incubated for 10 min on the ice. The cell pellet was collected by centrifugation at 1200×g for 5 min. Cells were lysed with ice-cold hypotonic lysis buffer (10 mM Hepes-NaOH [pH 7.5], 10 mM NaCl, 2 mM MgCl_2_, 1 mM EDTA, 1μg of leupeptin/ml, 1 μg of aproptinin/ml, 50 μg of PMSF/ml, 1 mM Na_3_VO_4_, 1 mM NaF, 0.3% NP-40 and 0.2% sodium deoxycholate), shaking vigorously 30 s. The nuclei pellet was collected by centrifugation at 2800×g for 5 min, supernatant reserved for cytoplasmic fraction. The nuclear pellet was added to hyper saline lysis buffer (10 mM Tris [pH 7.2], 0.5 M NaCl, 50 mM MgCl_2_, 0.1 mM CaCl_2_, 20 U RNase inhibitor, 150 U DNase I) at room temperature for 10 min, then centrifuged at 12000×g for 10 min. The pellet was further extracted in extraction buffer (10 mM Tris [pH 7.2], 10 mM NaCl, 10 mM EDTA), and centrifuged at 12000×g for 10 min.

***Statistical analysis.*** Student's test was used to calculate the statistical significance of the experimental data. The level of significance was set as *P≤0.05, **P≤0.01 and ***P≤0.001.

## Results

### Cellular localization of RPL15

**Figure 1:**
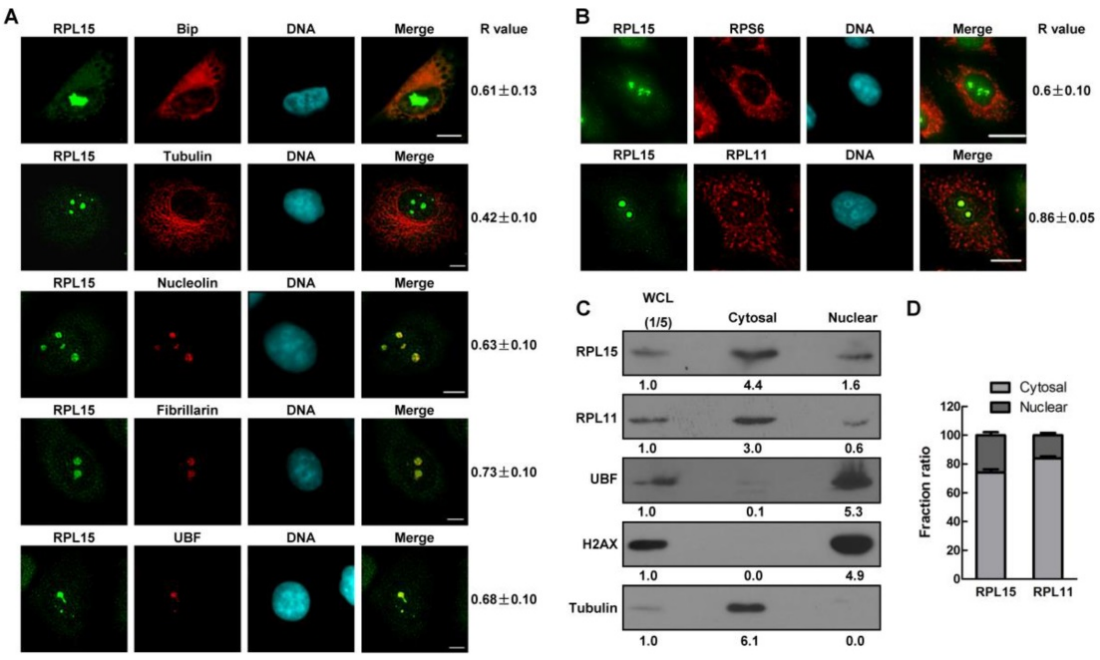
** Characterization of ribosomal protein RPL15. (A)** Immunofluorescence analysis of HeLa cells with rabbit α-RPL15 and mouse α-Bip, anti-α-tubulin, α-nucleolin, α-fibrillarin or α-UBF. DNA was visualized by DAPI staining. R values were obtained as described (see Materials and Methods). Scale bars, 10 μm. **(B)** Immunofluorescence analysis of HeLa cells with rabbit α-RPL15 and mouse α-RPL11 or mouse α-RPS6 and DAPI. Scale bars, 10 μm. R values were obtained as described in **(A)**. **(C)** Nuclear or cytoplasmic lysates from HeLa cells were extracted and subjected to immunoblotting by indicated antibodies. The density of each protein in each component was quantitated against the level of WCL (1/5 of total). WCL: whole cell lysate. **(D)** Histograms represented average proportion of RPL15 or RPL11 in Nuclear or cytoplasmic component relative to WCL in **(C).** All experiments were performed in triplicate. Data are shown as mean ± standard deviations.

RPL15 is a 60S large subunit protein, sequence analysis showed that RPL15 was conserved across many species (Figure S1A). To explore the role of RPL15 in human colon carcinogenesis, we generated rabbit polyclonal anti-RPL15 antibodies (α-RPL15). To verify the specificity of α-RPL15, we examined subcellular localization of RPL15 using immunofluorescence assay. RPL15 was co-stained with Bip (a rough ER marker), α-tubulin (cytoskeletal microtubules), nucleolin (nucleolar granular component marker), fibrillarin (nucleolar dense fibrillar component marker) and UBF (nucleolar fibrillar center marker). It was found that RPL15 dispersed localized in cytoplasm and nucleoplasm, and focused localized at nucleoli (Figure 1A). Co-fluorescence imaging and Pearson correlation coefficient (R value) analysis revealed that RPL15 was colocalized with Bip, but not with α-tubulin in cytoplasm, and colocalized with nucleolin, fibrillarin, and UBF in the nucleoli, which is coincident with the function of RPL15 in ribosome assembly and rRNA processing as previous reported^12^, ^24^. Furthermore, the endogenous RPL15 localization was consistent with exogenous expressed GFP-RPL15, but not GFP protein (Figure S1B).

We further compared the localization of RPL15 with other ribosomal proteins, RPL11 and RPS6. Immunofluorescence results showed that RPL15 was co-localized with RPL11 or RPS6 (Figure 1B). The R value of RPL15/RPL11 or RPL15/RPS6 was 0.86±0.05 or 0.6±0.10 (Figure 1B). Furthermore, it was observed that RPL15 was more focused on nucleolus than RPL11 or RPS6. To verify this phenomenon, immunoblotting analysis of subcellular cytoplasmic and nuclear fractions demonstrated that both RPL15 and RPL11 could be detected in cytoplasmic and nuclear fractions, but the percentage of nuclear localized RPL15 was significantly more than that of RPL11 (Figure 1C and D). Taken together, these results demonstrated that ribosomal protein RPL15 was dispersed in cytoplasm and nucleoplasm, and concentrated in the nucleolus in human cells.

### RPL15 is required for maintaining normal nucleolar structure

**Figure 2:**
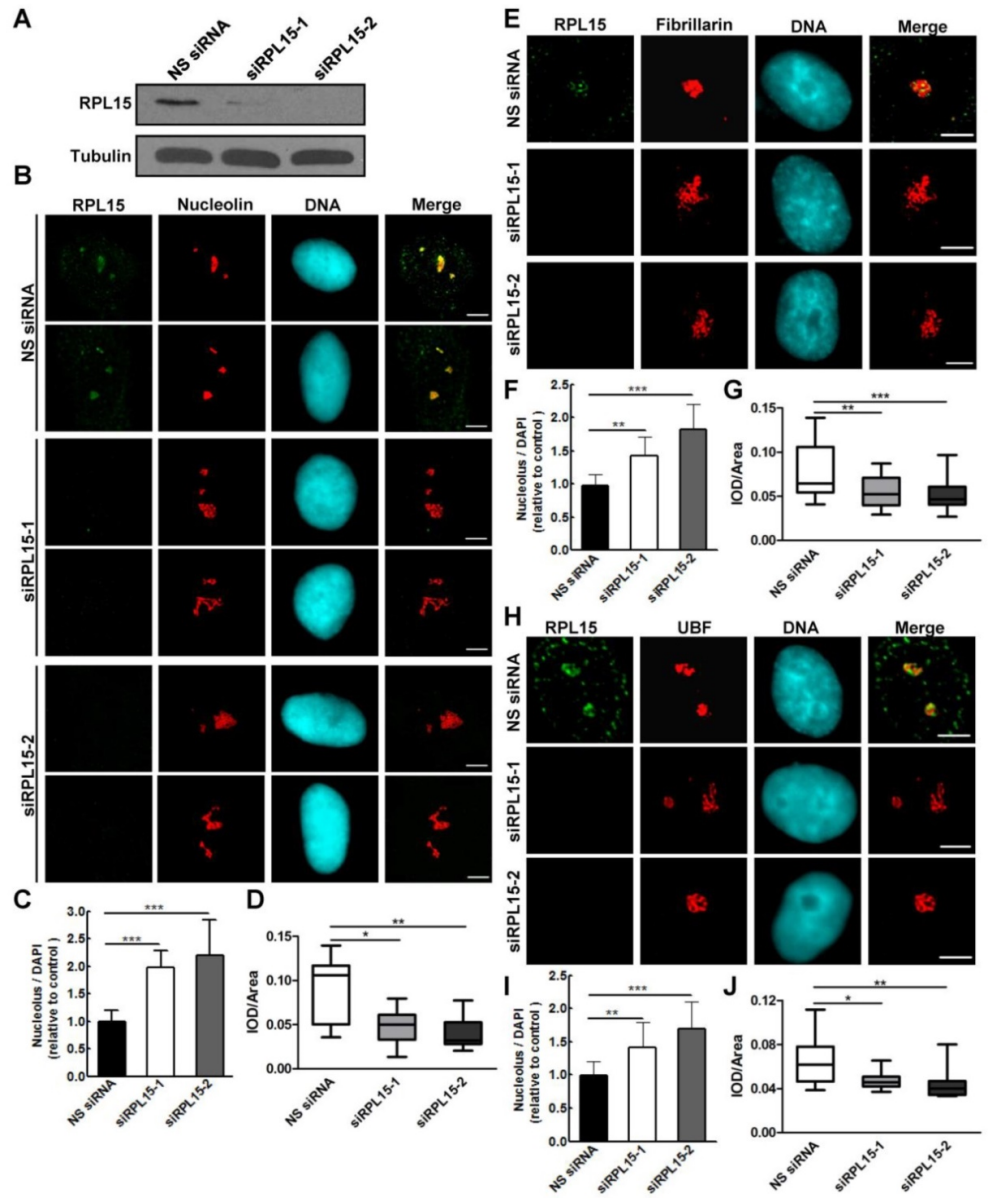
** The effects of RPL15 depletion on maintaining of nucleolar structure. (A)** HeLa cells were transfected with nonsense (NS) siRNA or RPL15 siRNA for 48 h and cell lysates were immunoblotted with α-RPL15 and anti-α-Tubulin antibody. **(B)** HeLa cells grown on coverslips were transfected with NS siRNA or RPL15 siRNA for 48 h, fixed and immunostained with α-RPL15 and α-nucleolin, DNA was visualized by DAPI staining. Scale bars, 10 μm. **(C-D)** Immunofluorescent area and intensity of nucleolin or DAPI in **(B)** in 20 cells were determined by Image-Pro Plus 7.0. The ratio of nucleolin area to DAPI against controls **(C)** and immunofluorescent density (IOD/area)** (D)** were calculated. **(E)** Cells treated as **(B)** were fixed and immunostained with α-RPL15 and α-fibrillarin, DNA was visualized by DAPI staining. Scale bars, 10 μm. **(F-G)** Immunofluorescent area and intensity of fibrillarin or DAPI in **(E)** in 20 cells were determined. The ratio of fibrillarin area to DAPI against controls** (F)** and immunofluorescent density (IOD/area) **(G)** were calculated. **(H)** Cells treated as **(B)** were fixed and immunostained with α-RPL15 and α-UBF, DNA was visualized by DAPI staining. Scale bars, 10 μm. **(I-J)** Immunofluorescent area and intensity of UBF or DAPI in **(H)** in 20 cells were determined. The ratio of UBF area to DAPI against controls **(I)** and immunofluorescent density (IOD/area) **(J)** were calculated. Results represent means ± standard deviations of five independent experiments *P<0.05; ** P<0.01; *** P<0.001.

Nucleolus is a dynamic structure that responsible for ribosomal RNA synthesis and nascent ribosome assembly^34^. RPL15 localizes at nucleolus, so we want to determine the function of RPL15 in nucleolus. We ablated RPL15 in HeLa cells using specific RPL15 small interfering RNAs (siRNAs). Transfection of RPL15 siRNA (siRPL15-1 or -2), but not nonsense siRNA (NS siRNA), significantly reduced endogenous protein levels of RPL15 (Figure 2A). Immunofluorescence of nucleolin in control or RPL15 depleted cells was determined. As shown in Figure 2B, in contrast to control cells, cells depleted of RPL15 showed an increased nucleolin area in nucleus. To characterize nucleolar morphology defects quantitatively, we developed a specific image-processing algorithm. Briefly, we first measured the area of observed nucleoli and nucleus of each cell on the basis of nucleolin and nuclear stain (4,6-diamidino-2-phenylindole (DAPI) signal). Then, the ratio of nucleolar area relative to nuclear area was determined. The results showed that depletion of RPL15 induced expanded nucleoli in nucleus when compared with controls (Figure 2C). Meanwhile, the fluorescent density (IOD/Area) of nucleolin was notablely decreased after RPL15 depletion (Figure 2D).

It was reported that RPL15 not only participated in ribosome assembly, but also involved in rRNA processing^12^, ^24^. Thus, we speculated that RPL15 depletion not only induces the alteration of peripheral nucleolar structure (granular component region) but also causes defect in intrinsic nucleolar structure (dense fibrillar component or fibrillar center region). To explore these possibilities, we detected the alteration of fibrillarin or UBF staining after RPL15 depletion. Similar to nucleolin, RPL15 depletion also caused an increase in fluorescent area and a decrease in fluorescent density of fibrillarin (Figure 2E-G) or UBF (Figure 2H-J), which indicated that whole nucleolar structure become incompact after RPL15 depletion. Taken together, these results suggested that RPL15 was required for normal nucleolar structure maintenance.

### RPL15 is required for pre-60S ribosomal subunit biogenesis

**Figure 3:**
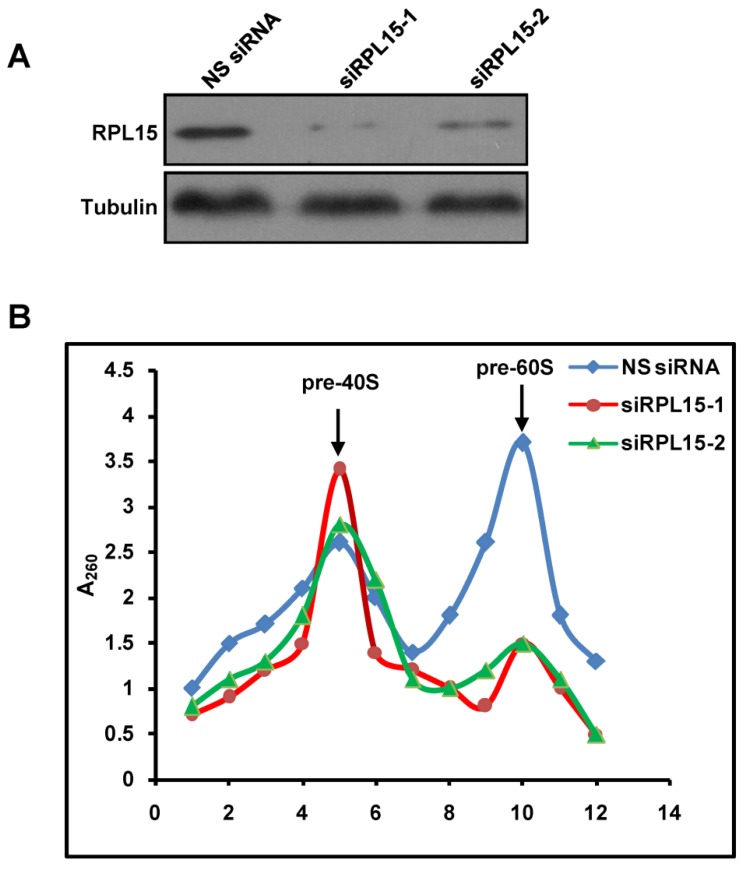
** Ablation of RPL15 compromises ribosome biogenesis of pre-60S particles.** (A) HeLa cells were transfected with nonsense (NS) siRNA or RPL15 siRNA for 48 h and cell lysates were immunoblotted with α-RPL15 and anti-α-Tubulin antibody. **(B)** Cells were treated as in **(A),** and nuclear extracts were prepared from HeLa cells and fractionized on 10% to 40% sucrose density gradient. The absorbance at 260 nm (A_260_) of each fraction was profiled and the positions of pre-ribosomal subunits were indicated.

Previous study demonstrated that RPL15 participated in rRNA processing at ITS1 site, and rRNAs required for both 40S and 60S ribosomal subunits assembly were impaired after RPL15 deletion^12^. However, the effect of RPL15 depletion on ribosomal subunits assembly is unclear. Together with our results that RPL15 participated in maintaining nucleolar structure, we speculated that RPL15 regulates pre-ribosome formation. To this end, we ablated expression of RPL15 by RPL15 siRNA and analyzed assembly of pre-ribosomal subunits using a sucrose gradient by ultracentrifugation. As shown in Figure 3, sucrose gradient analysis indicated that, when compared to control, ablation of RPL15 resulted in a significant reduction of pre-60S ribosomal subunits but an increase of pre-40S ribosomal subunits in HeLa cells. This result indicated that RPL15 was required for regulating in pre-60S ribosomal subunit biogenesis.

### RPL15 is overexpressed in colon cancer

**Figure 4:**
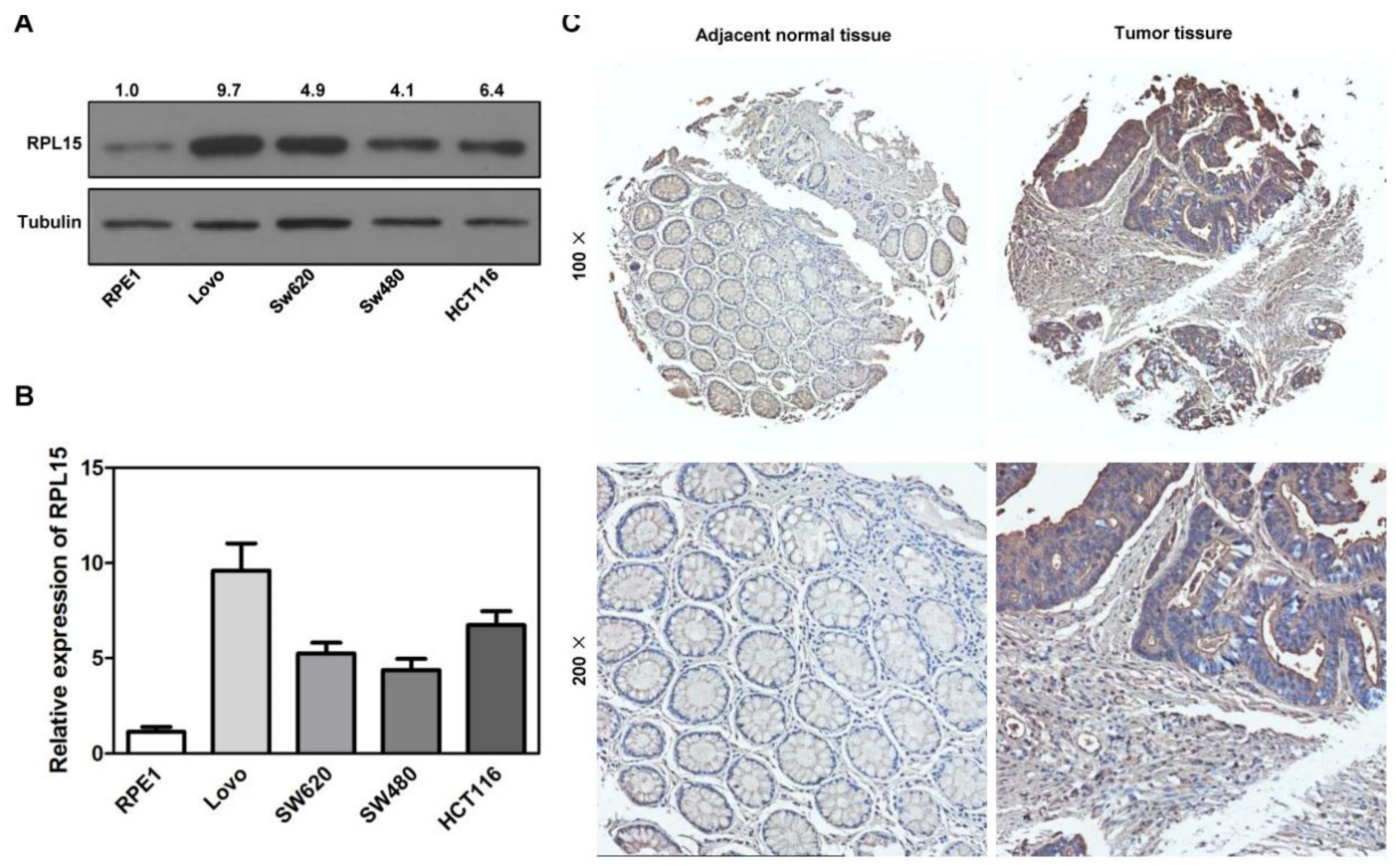
**Expression of RPL15 is upregulated in colon cancer. (A)** The expression level of RPL15 was detected by immunoblotting in four colon cancer cell lines and a non-transformed epithelial cell line (RPE1). The density of each protein in each plane was quantitated. **(B)** quantification of the relative protein level of RPL15 in four colon cancer cell lines and non-transformed epithelial RPE1 cell. Data represent means ± standard deviations of three independent experiments. **(C)** RPL15 immunostaining in TMAs are shown. Note: top panel, magnification ×100; bottom panel, magnification ×200.

Ribosome is responsible for cellular protein synthesis, and large bodies of evidences demonstrated that ribosomal dysfunction lead to various diseases, such as cancer, anemia, etc. It has been reported that RPL15 was depleted in DBA and played an intriguing role in carcinogenesis^12^, ^25^-^27^, ^35^. We examined expression levels of RPL15 in public available human cancer microarray studies using the ONCOMINE database analysis, and the result revealed that RPL15 was upregulated in colon cancer tissues when compared with normal tissues (Figure S2A). To investigate the critical role of RPL15 in colon carcinogenesis, we first detected the expression of RPL15 in four colon cancer cell lines using western blot. In comparison with a non-transformed epithelial cell line (RPE1), RPL15 protein expression was upregulated in all four colon cancer cell lines (LoVo, SW480, SW620 and HCT116) (Figure 4A and B). Subsequently, we collected 25 pairs of colon cancer tissue and corresponding adjacent non-cancerous tissue and evaluated the RPL15 expression using immunohistochemistry (IHC) (Figure 4C). We found that 56.5% (13 of 25) colon cancer tissue showed a higher level of RPL15 compared with the corresponding adjacent non-cancerous tissue. Statistical analysis showed that RPL15 expression in colon cancer was positively correlated with tumor stage (P<0.01), and no significant association was found between RPL15 expression and other parameters (Table 1). Furthermore, relative expression of RPL15 mRNA and protein in these tissues were quantified by analyzing the results of realtime PCR and immunoblotting. The results were consistent with IHC and showed that there is about a 1.8 fold higher level of RPL15 expression in total cancer tissue than that of adjacent tissue (Figure S2B-F). These results suggested that RPL15 was overexpressed in colon cancer and involved in colon carcinogenesis.

### RPL15 dependent ribosome stress induces apoptosis in colon cancer cells, but cell cycle arrest in non-transformed epithelial cells

**Figure 5:**
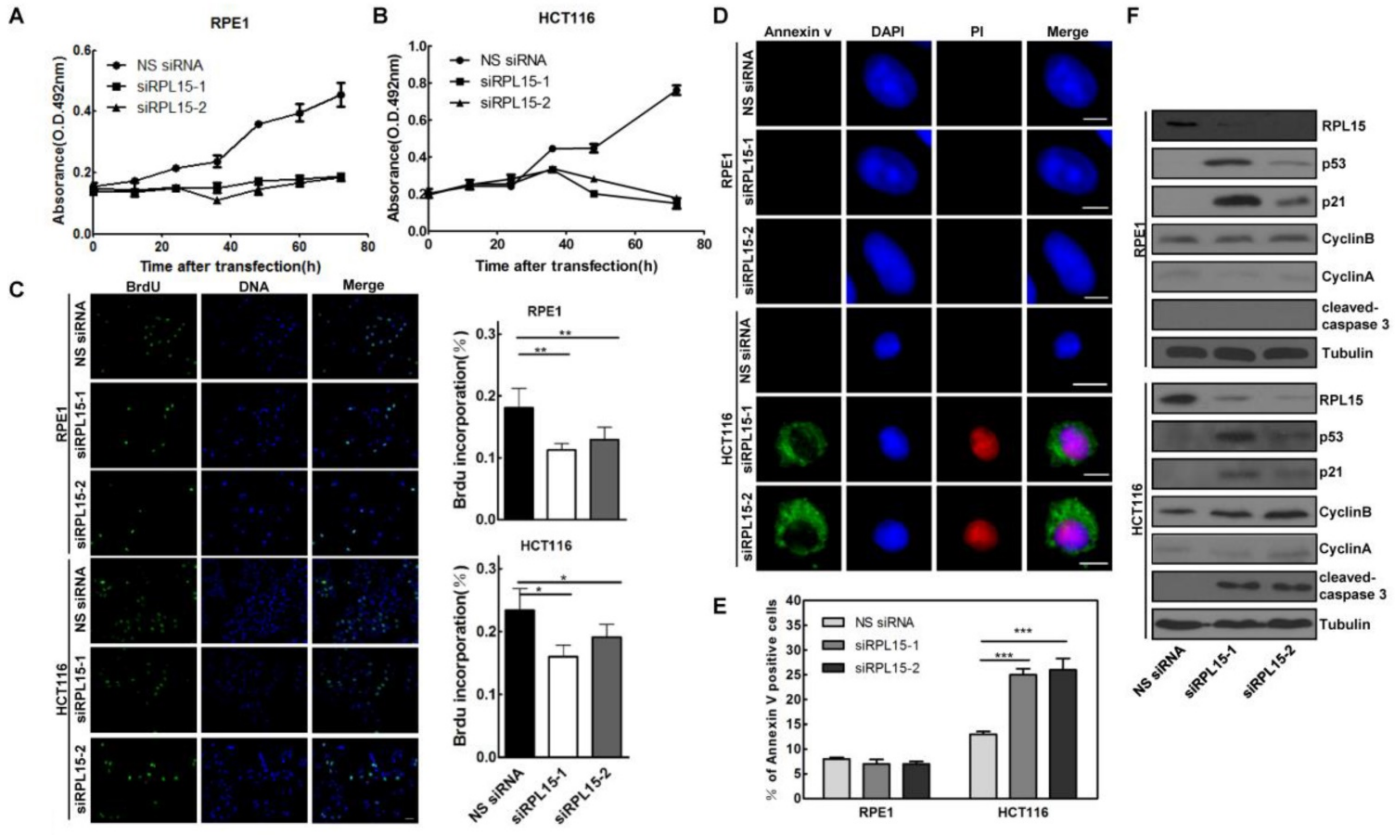
** The effects of RPL15 ablation on cell proliferation and viability in various human cells.** (A-B) RPE1 **(A)** and HCT116 **(B)** were transfected with NS siRNA or RPL15 siRNA and cell proliferation was determined by MTT assay at indicated times. Results represent means ± standard deviations of five independent experiments. **(C)** Cells transfected with NS siRNA or RPL15 siRNA for 48 h were labeled with 10 mM BrdU (1 h). The cells were fixed and immunostained with mouse α-BrdU. DNA was visualized by DAPI staining. Histograms represented the mean percentage of BrdU incorporation in indicated human cells depleted of RPL15. *P<0.05 and **P<0.01. **(D)** Cells were transfected with NS siRNA or RPL15 siRNA for 48 h. The cells were fixed and stained with Annexin V and PI. DNA was visualized by DAPI staining. Scale bars, 10 μm. **(E)** Histograms represented the mean percentage of Annexin V-positive cell in indicated human cells depleted of RPL15. ***P<0.001. **(F)** Immunoblotting to detect the effect of RPL15 depletion on cell cycle or apoptosis related proteins in indicated human cells. Cells were transfected with NS siRNA or RPL15 siRNA for 48 h. Whole cell lysates were immunoblotted with indicated antibodies.

Our results showed that RPL15 was overexpressed in colon cancer tissue and its expression was associated with the progression of colon cancer, we next sought to detect the biological function of RPL15 in colon cancer. Human colon cancer cell line, HCT116, and a non-transformed epithelial cell line, RPE1, were transiently transfected with RPL15 siRNAs or NS siRNA. MTT assay revealed that depletion of RPL15 in HCT116 or RPE1 resulted in a significant inhibition of cell proliferation as compared with controls (Figure 5A and B). However, while RPE1 cells depleted of RPL15 exhibited a 58.9% decrease in proliferation rate, HCT116 cells depleted of RPL15 displayed complete inhibition of cell proliferation, and viable cells were decreased at 72 h after RPL15 siRNA transfection, which suggested that RPL15 depletion caused cell death in HCT116 cells. To further verify the cell proliferative effects of RPL15 ablation, we monitored S-phase progression by BrdU incorporation assay in HCT116 or RPE1 cells treated with NS or RPL15 siRNAs. As shown in Figure 5C, when compared with controls, depletion of RPL15 caused significant reduction of BrdU incorporation in HCT116 or RPE1 cells, which indicated decreased proliferative capacity of these cells after RPL15 depletion.

Next, we detected the effect of RPL15 depletion on cell cycle using FACS analysis, and the results showed that, when compared with controls, depletion of RPL15 in RPE1 cells resulted in an increased accumulation of cells in G1 to G1/S phase whereas depletion of RPL15 in HCT116 cells caused an increase of cells in sub-G1 phase (cell death) (Figure S3). To verify the cell death after RPL15 depletion, Annexin V/PI staining assay was performed, and the results showed that, when compared with controls, depletion of RPL15 in HCT116 cells, but not RPE1 cells, induced apoptotic markers annexin V positive cell staining (Figure 5D and E). Consistently, immunoblotting demonstrated that depletion of RPL15 in HCT116 cells, but not RPE1 cells, induced caspase 3 activation (Figure 5F). In addition, consistent with other ribosomal proteins, depletion of RPL15 induced ribosome stress also caused accumulations of p53 and p53 downstream target, CDK inhibitor p21 in RPE1 or HCT116 cells (Figure 5F)^36^, ^37^. These results indicated that RPL15 dependent ribosome stress induced cell cycle arrest at G1-G1/S phase in non-transformed RPE1 cells, but resulted in apoptosis in HCT116 colon cancer cells.

## Discussion

In this study, we studied the role of RPL15 in colon cancer carcinogenesis. We found that RPL15 was overexpressed in colon cancer cell lines and cancer tissue, and elevated expression of RPL15 in colon cancer tissues was closely correlated with clinicopathological characteristics in patients. Depletion of RPL15 induced enlarged nucleolar structure and compromised pre-60S ribosome biogenesis. We further demonstrated that RPL15 dependent ribosome stress resulted in cell cycle arrest at G1-G1/S phase in non-transformed RPE1 cells, but apoptosis in colon cancer HCT116 cells. These results suggested that RPL15 could be a potential biomarker for colon cancer targeted therapy.

RPL15 is a 60S ribosomal protein, and it was deemed to play a role in ribosome assembly and protein synthesis in past years. Indeed, our results demonstrated that RPL15 dispersed localized at cytoplasm and colocalized with Bip, a rough ER marker, which suggested that RPL15 participates in ribosomal assembly and involves in protein translation. However, other study and our results demonstrated that, compared with other RPs, the nucleolar localization of RPL15 was more distinct, which indicated that RPL15 takes part in some other process in nucleolus^38^. Previously, Michael Landowski et al. found that RPL15 participated in rRNA processing at ITS1 site, and depletion of RPL15 resulted in alteration of rRNA precursors required for both 60S and 40S ribosome biogenesis^12^. Consistent with the results, we demonstrated that ablation of RPL15 induced a decrease of pre-60S ribosomal particle and an increase of pre-40S ribosomal particle. Moreover, we also found that RPL15 depletion enlarged the nucleolus, and the alteration of nucleolar structure may due to compromised rRNA processing and ribosome assembly.

Large bodies of evidences have demonstrated that ribosomal proteins involve in multiple human diseases. It was found that RPL15 exists deletion in DBA, indicates that RPL15 may play a critical role in pathogenesis of the syndrome^12^. However, the expression of RPL15 appears to be different dependent on types of cancer^25^-^27^, ^35^. Here, we demonstrated that the expression of RPL15 in colon cancer tissues was higher than that in adjacent normal tissues. Furthermore, the expression level of RPL15 was associated with colon cancer progression. Depletion of RPL15, in contrast to non-transformed RPE1 cells, resulted in specific apoptosis in colon cancer cells. However, the mechanisms by which RPL15 depletion resulted in specific apoptosis in colon cancer cells are unclear and need to further investigate.

In conclusion, we demonstrated that RPL15 had an important role in nucleolar structure maintenance and ribosome biogenesis. RPL15 was upregulated in colon cancer tissue and closely associated with colon cancer progression. Furthermore, RPL15 dependent ribosome stress induced specific cell apoptosis in colon cancer cells. Therefore, RPL15 could be a prospective target for therapy of colon cancer.

## Supplementary Material

Supplementary figures and tables.

## Acknowledge

This work was supported by CAMS Innovation Fund for Medical Sciences (CIFMS) (2016-12M-1-001), the National Natural Science Foundation of China (81575685 and 81703013), Tianjin Science and Technology Project (17YFCZZC00540 and 18YFZCSY00100), Tianjin Science & Technology Fund Planning Project of colleges and universities (2017KJ120) and Tianjin 131 Creative Talents Cultivation project (135305QS17).

### Author Contributions statement

All authors participated in the design, interpretation of the studies and analysis of the data and review of the manuscript. Z.D., C.Z. and W. J. designed research;, Z.D., H.J, and S.L. performed research; Y.W. contributed reagents; Z.D., C.Z. and W.J. analyzed data; Z.D. wrote the paper.

### Disclaimers

The views expressed in the submitted article are his or her own and not an official position of the institution or funder.

## Figures and Tables

**Table 1:** Clinical profiles and correlation between the clinicopathologic features and expression of RPL15

Association between RPL15 in colon cancer and patient characteristics					
	Total (n=25)	Positive (n=13)	Negative (n=12)	X^2^	p value (positive vs negative)
Gender				0.962	NS
Female	10	4	6		
Male	15	9	6		
Age				0.037	NS
≤60	13	7	6		
>60	12	6	6		
Anemia				0.987	NS
Y	12	5	7		
N	13	8	5		
CEA				1.066	NS
≥5	11	7	4		
<5	14	6	8		
TNM Stage				7.354	0.01*
Stage I-II	8	1	7		
Stage III-IV	17	12	5		
Differentiation				1.708	NS
Low	2	1	1		
Middle+High	23	12	11		

Positive: adjacent tissue> carcinoma; Negative: adjacent tissue≤ carcinoma
