# Supplementary material for: Ribosomal Protein L15 is involved in Colon Carcinogenesis
Source: Int J Med Sci. 2019 Aug 6;16(8):1132–41. doi: 10.7150/ijms.34386 (PMC6743284; doi:10.7150/ijms.34386)
Supplement: Supplementary file 1 — Supplementary figures and tables. [file ijmsv16p1132s1.pdf]

Supplemental Figure Legends:

Figure S1. The phylogenetic tree of RPL15 in eukaryotes and localization of exogenous expressed RPL15. (A) Shown is the phylogenetic tree of RPL15 proteins in various eukaryotic organisms. (B) HeLa cells grown on coverslips were transfected with plasmids of pEGFP or pEGFP-RPL15 for 24 h. The cells were fixed and stained with mouse α-nucleolin and DAPI. Scale bars, 10 μm. R values were obtained as described (see Materials and Methods).

Figure S2. RPL15 is upregulated in colon cancer tissue. (A) Data analysis was performed with the ONCOMINE 3.0 database using standard settings (www.oncomine.org). Class 1: RPL15 mRNA expression in normal colon tissues. Class 2: RPL15 mRNA expression in normal rectum tissues. Class 3: RPL15 mRNA expression in colon cancer tissues. (B) RPL15 mRNA is upregulated in tumor tissues obtained from colon cancer patients. (C) The average relative expression of pre-45s rRNA in both adjacent normal and tumor tissues. (D) Immunoblotting to detect the expression of RPL15 protein in colon cancer tissues and their adjacent normal tissues. The density of each protein in each component was quantitated and relative expression of RPL15 was analyzed. (E) The relative expression of RPL15 protein in colon cancer tissues and their adjacent normal tissues. (F) The average relative expression of RPL15 protein in both adjacent normal and tumor tissues. colon cancer tissues (T), adjacent normal tissues (N).

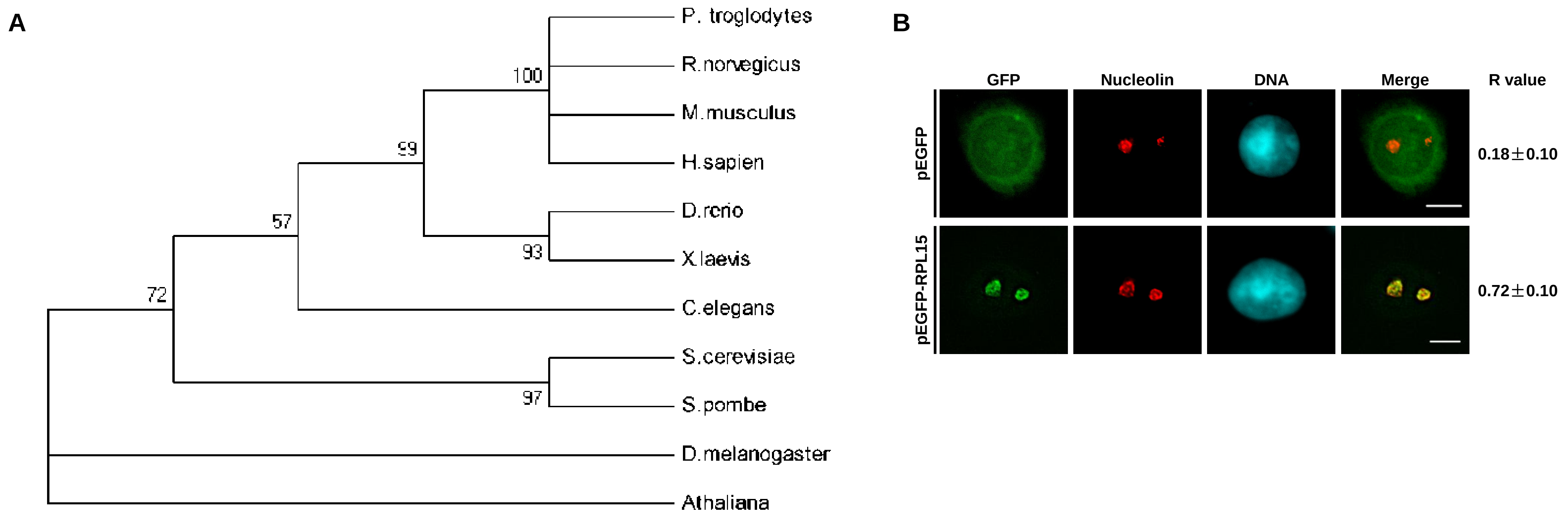

Figure S1

Dong ZX et al.

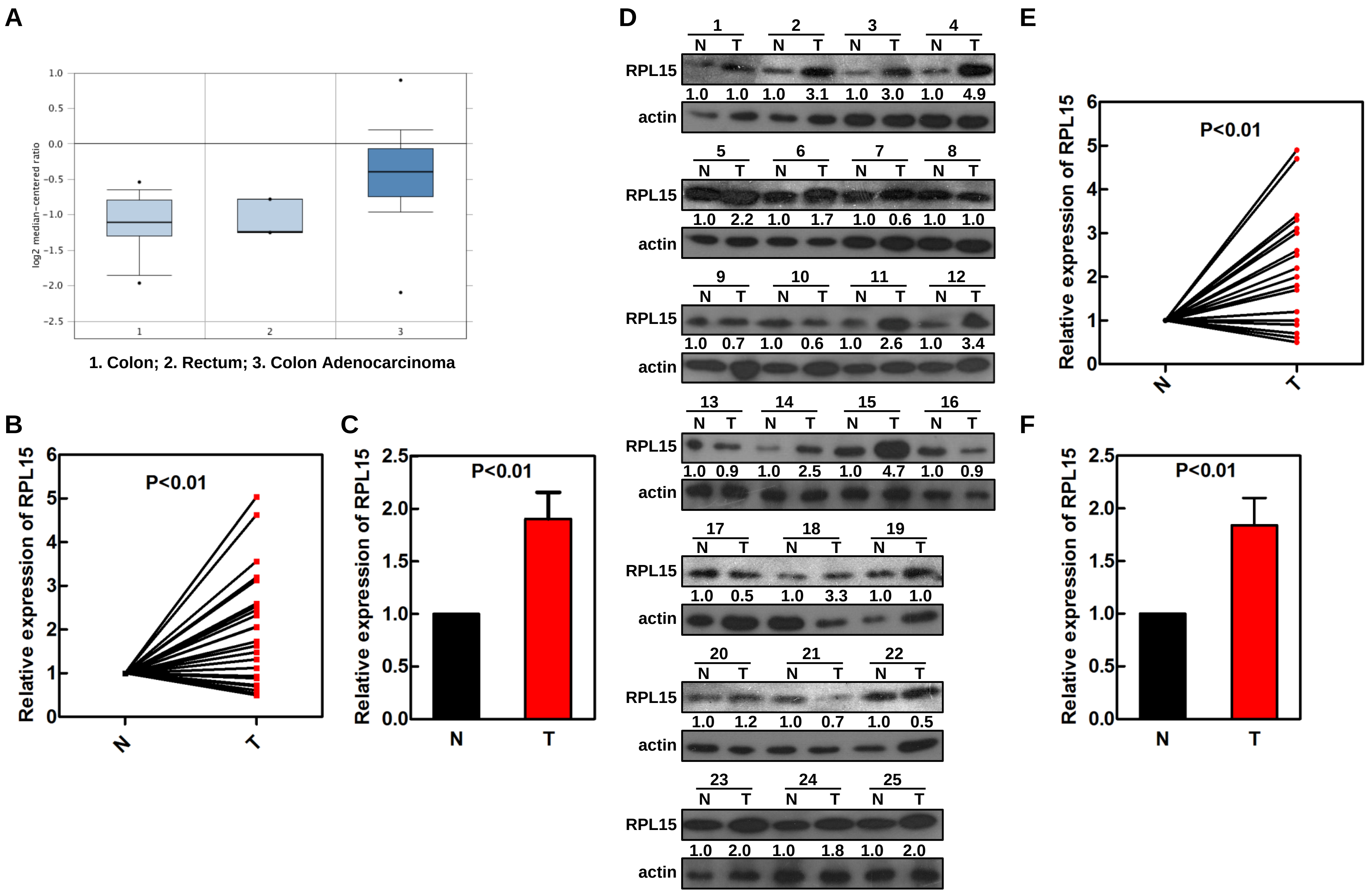

Figure S2

Dong ZX et al.

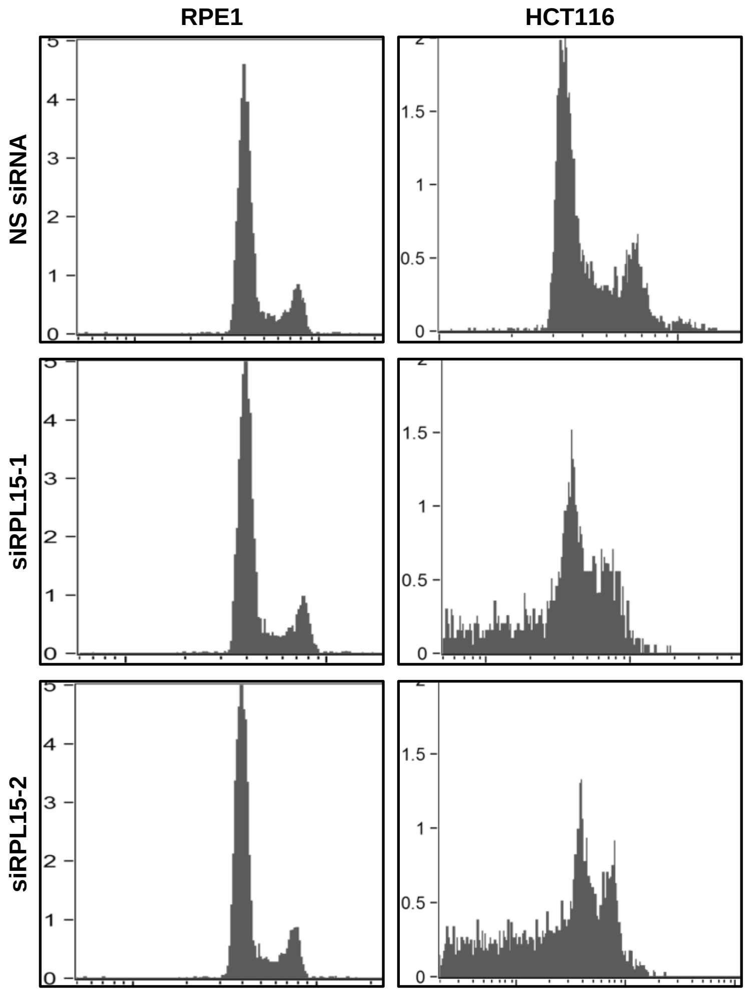

Figure S3

Dong ZX et al.
